# SAK3 confers neuroprotection in the neurodegeneration model of X-linked Dystonia-Parkinsonism

**DOI:** 10.21203/rs.3.rs-4068432/v1

**Published:** 2024-04-25

**Authors:** Shivani Aryal, Shawei Chen, Kyle F Burbach, Yan Yang, Lucia S Capano, Woo Kyung Kim, D. Cristopher Bragg, Andrew Yoo

## Abstract

Background X-linked Dystonia-Parkinsonism(XDP) is an adult-onset neurodegenerative disorder that results in the loss of striatal medium spiny neurons (MSNs). XDP is associated with disease-specific mutations in and around the *TAF1* gene. This study highlights the utility of directly reprogrammed MSNs from fibroblasts of affected XDP individuals as a platform that captures cellular and epigenetic phenotypes associated with XDP-related neurodegeneration. In addition, the current study demonstrates the neuroprotective effect of SAK3 currently tested in other neurodegenerative diseases. Methods XDP fibroblasts from three independent patients as well as age- and sex-matched control fibroblasts were used to generate MSNs by direct neuronal reprogramming using miRNA-9/9*-124 and thetranscription factors *CTIP2* , *DLX1* -P2A- *DLX2* , and *MYT1L* . Neuronal death, DNA damage, and mitochondrial health assays were carried out to assess the neurodegenerative state of directly reprogrammed MSNs from XDP patients (XDP-MSNs). RNA sequencing and ATAC sequencing were performed to infer changes in the transcriptomic and chromatin landscapesof XDP-MSNs compared to those of control MSNs (Ctrl-MSNs). Results Our results show that XDP patient fibroblasts can be successfully reprogrammed into MSNs and XDP-MSNs display several degenerative phenotypes, including neuronal death, DNA damage, and mitochondrial dysfunction, compared to Ctrl-MSNs reprogrammed from age- and sex-matched control individuals’ fibroblasts. In addition, XDP-MSNs showed increased vulnerability to TNFα -toxicity compared to Ctrl-MSNs. To dissect the altered cellular state in XDP-MSNs, we conducted transcriptomic and chromatin accessibility analyses using RNA- and ATAC-seq. Our results indicate that pathways related to neuronal function, calcium signaling, and genes related to other neurodegenerative diseases are commonly altered in XDP-MSNs from multiple patients. Interestingly, we found that SAK3, a T-type calcium channel activator, that may have therapeutic values in other neurodegenerative disorders, protected XDP-MSNs from neuronal death. Notably, we found that SAK3-mediated alleviation of neurodegeneration in XDP-MSNs was accompanied by gene expression changes toward Ctrl-MSNs.

## Background

X-linked dystonia-parkinsonism (XDP) is a Mendelian adult-onset neurodegenerative disorder that was initially identified in individuals from the island of Panay in the Philippines (1). The pathogenic gene lesion has been identified as a disease-specific insertion of a SINE-VNTR-Alu (SVA)-type retrotransposon within an intron of *TAF1* (2–4). The SVA contains a hexameric DNA repeat expansion $(CCCTCT)_n$, the length of which is polymorphic among patients and inversely correlated to the age at disease onset (3, 5). The clinical disease manifestations of XDP are heterogeneous, with some individuals exhibiting initial dystonia that combines with or is replaced by parkinsonism over time versus others in which parkinsonian features are the predominant phenotype (6–8). The neuroanatomical substrates of these motor deficits have not been fully defined, but neuroimaging studies in patients and neuropathological analyses of postmortem brain tissue have collectively shown that XDP involves progressive atrophy of the neostriatum that may be due primarily to the loss of medium spiny neurons (MSNs) (9–14). However, the exact mechanisms and pathways underlying this degeneration remain largely unknown.

Here, we describe cellular phenotypes associated with XDP in MSNs directly reprogrammed from XDP patient-derived fibroblasts as a model system to understand the molecular underpinnings of MSN degeneration in XDP (14, 15). Direct neuronal reprogramming offers several advantages to studying adult-onset pathological features since directly reprogrammed neurons mimic the chronological age of fibroblast donors as assessed by DNA-methylation-based epigenetic age contrasting neurons differentiated from induced pluripotent stem cells representing an embryonic state (16). Direct neuronal reprogramming method has been used to gain insights into adult-onset neurodegenerative states in other diseases, such as Huntington's disease and primary tauopathies (17–23). In addition, while XDP is strictly an inherited disorder, aging till contributes to the disease onset as in other neurodegenerative disorders (24) as XDP patients remain asymptomatic until they reach middle age. Cellular hallmarks of aging include changes in gene expression that are involved in DNA damage responses, synaptic transmission, mitochondrial function, insulin signaling, and lipid metabolism (16, 24, 25). In the modeling of adult-onset inherited neurodegenerative disorders such as Huntington's disease, these age-associated changes become exacerbated contributing to the onset of neurodegeneration (20, 24, 26, 27).

In the current study, we used MSNs directly reprogrammed from XDP patient fibroblasts using MSN-reprogramming effectors including the neurogenic microRNAs (miRNAs), miR-9/9*, and miR-124 (miR-9/9*-124), and striatum-enriched transcription factors *CTIP2, DLX1-2* and *MYT1L* that guide the miRNA-induced conversion towards MSNs (17, 18, 28, 29). Directly reprogrammed XDP-MSNs recapitulate neurodegenerative phenotypes that resemble features detected in the brains of XDP individuals and not present in MSNs reprogrammed from control adult fibroblasts. Interestingly, when comparing XDP vs. control fibroblasts, there were minimal differentially expressed genes (DEGs) but comparison of XDP vs control MSNs resulted in more than 500 significant DEGs that arose as a result of cells adopting the MSN identity. These DEGs were enriched for several genetic pathways including calcium dysregulation. We then tested whether SAK3, a T-type calcium channel agonist (30), would alleviate neurodegeneration in XDP-MSNs as SAK3 has been suggested to be neuroprotective in other neurodegenerative diseases such as Alzheimer's Disease and Lewy body dementia (30–32), and also shown to rescue pathology caused by *Taf1* knockdown in rodent brains (33, 34). We found that the neurodegeneration phenotype in XDP-MSNs could be mitigated by treatment with SAK3, which correlates with a significant portion of DEGs in XDP-MSNs reversing toward Ctrl-MSNs. Overall, our study shows that directly reprogrammed XDP-MSNs models the adult-onset neurodegeneration of XDP characterized by gene expression and chromatin dysregulation and demonstrate the protective effect of SAK3 in XDP patient-derived neurons.

## Methods

1. Fibroblast lines

Adult dermal fibroblasts from symptomatic patients with XDP and healthy controls were acquired from the CCXDP and NINDS repositories (Supp Table 1) (4). Primary fibroblasts were cultured in fibroblast culture media (15% FBS in DMEM) until ready for reprogramming experiments.

2. Lentivirus preparation for neuronal reprogramming

Plasmids for virus preparation are publicly available at Addgene as pTight-9-124-BclxL (60857), rtTA-N144 (66810), pm*CTIP2*-N106 (66808), ph*MYT1L*-N174 (66809), ph*DLX1*-N174 (60859) and ph*DLX2*-N174 (60860) (*DLX1* and *DLX2* were cloned together with the self-cleaving P2A motif and used in the reprogramming experiments in this study).

Lentivirus was made by transfecting 293LE cells with 1.5 μg of pMD2. G, 4.5 μg of psPAX2, 6 μg of lentiviral plasmid, 600 μL of Opti-MEM and 48 μL of PEI solution. Individual plasmids were made into lentiviruses, but they were pooled together during concentration. Lentivirus supernatant was collected and filtered through a 0.45 μm PES membrane. A LentiX concentrator was added to the supernatant at a 1:4 ratio and left overnight at 4 ℃. The solution was spun down for 45 min at 1000 g and 4 ℃. The pellet was resuspended in 1/10th volume of PBS. 7 ml of 10% sucrose solution was added to a high-speed centrifuge tube. The virus solution in PBS was carefully layered on top of the sucrose solution and centrifuged for 2 hours at 70,000 g at 4 ℃. The pellet was resuspended in 1/100th of the initial supernatant volume with 10% sucrose solution and frozen at -80 ℃ for future transduction.

3. Cell culture and reprogramming

Fibroblasts were first expanded in fibroblast culture medium (15% FBS in DMEM). They were split into 6-well plates for infection with lentivirus for the microRNAs and MSN-specific transcription factors. Reprogramming was performed using the previously described method until postinduction day (PID) 25–35. Briefly, fibroblasts were cultured until confluent. They were then plated onto 6-well tissue culture plates for transduction. Transduction was performed on Day 0 with lentiviruses for 9/9*-124, rtTA, ctip2, dlx1-p2a-dlx2 and myt1l and 1000X Polybrene. The cells were spinfected for 30 minutes. On day 1, the cells were refed with fibroblast culture medium and 1 μg/ml doxycycline. On day 3, the same feeding was performed but with the addition of the antibiotics puromycin (3 mg/ml), blasticidin (3 μg/μl) and G418 (300 μg/μl). Reprogramming cells were then replated onto polyornithine-, fibronectin- and laminin-coated coverslips on day 5. On day 6, blasticidin and G418 were added to the media for the second and last time. Cells were subsequently maintained in Neurobasal A (Gibco) media containing B-27 plus supplement and GlutaMAX supplemented with valproic acid (1 mM), dibutyl cAMP (200 mM), BDNF (10 ng ml − 1), NT-3 (10 ng ml − 1), RA (1 mM), ascorbic acid (200 μM) and RVC (RevitaCell Supplement, 1×) until day 13 of reprogramming. DOX treatment was cycled every 2 days, and half-volume feeding was performed every 4

days. From day 21, media with supplements except ascorbic acid (200 μM) and RVC (1×) were added in half-to-half volume.

4. Treatment with compounds

DNA damage rescue experiment in Fig. 2 was performed using KU-60019 (Millipore Sigma, Cat#SML1416). Recombinant human TNFα protein (R&D Systems, Catalog #: 210-TA) in multiple dosages was used for experiments in Figs. 2 and 5. SAK3 (Tocris, Cat# 6239) was used for experiments in Figs. 5 and 6.

5. Immunofluorescence staining and quantifications

Reprogrammed cells were fixed with 4% paraformaldehyde (Electron Microscopy Sciences, 15710) for 20 minutes at room temperature (RT) and then permeabilized with 0.2% Triton X-100 for 10 minutes at RT. Fixed cells were blocked with 5% BSA and 1% goat serum in PBS and incubated with primary antibodies at 4°C overnight. The cells were then incubated with secondary antibodies for 1 hour at RT. The primary and secondary antibodies used are detailed in Supplementary Table 2. After 1 wash with PBS, the cells were incubated with DAPI (Sigma—Aldrich, D-9542) for 5 minutes, followed by washing with PBS. Images were captured using a Leica SP5X white light laser confocal system with Leica Application Suite (LAS) Advanced Fluorescence 2.7.3.9723. All quantification was performed using the "surface" module in Imaris or manual counting.

6. Electrophysiology
Whole-cell patch-clamp recordings were performed on 7–8 weeks Medium Spiny Neurons (MSNs) after the cells were direct converted from human adult (young and old) fibroblasts. The MSNs including CTL and XDP were co-cultured with human astrocytes (Seong Won Lee, et al. 2023). The MSNs on coverslip were rapidly transported to the recording chamber of a Zeiss Axioskop Upright Microscope (Zeiss). Electrophysiological recordings were carried out in the extracellular solution modified (Victor, et al, 2014) consisting (in mM) of 140 NaCl, 3 KCl, 10 Glucose, 10 HEPES, 1.5 $CaCl_2$ and 1 $MgCl_2$ (pH adjusted to 7.3 with NaOH, Osmolarity was 295 ~ 310 mOsM). The recording was made at room temperature and was restricted to the first 2–3 hours following cell removal from the incubator environment. Electrode pipettes were pulled from borosilicate glass (World Precision Instruments) and typically the resistance ranged between 5–8 MΩ filled with intracellular solution (in mM) 125 K-Gluconate, 4 NaCl, 2 $MgCl_2$, 1 EGTA, 10 HEPES, 4 $Na_2$-ATP, 0.4 $Na_3$-GTP, 5 Creatine phosphate (pH adjusted to 7.5 with KOH, Osmolarity was 290–300 mOsM). Data was acquired using pCLAMP 10 software with multiClamp 700B amplifier and Digidata 1550 digitizer (Molecular Devices). Data was initially analyzed in Clampfit (Molecular Devices). Further analysis was done in GraphPad Prism 9.0 Software). Liquid junction potential was calculated to be 15.2 mV and corrected in calculating resting membrane potential according to previously published Methods (Victor 2017). In voltage –clamp mode, membrane potentials were typically kept at -70 mV, the Inward sodium Currents (INa) and the outward potassium currents (IK) were recorded with voltage steps ranging from − 20 mV to + 80 mV. In current-clamp mode, action potentials were elicited by injection of step currents that modulated membrane potential by holding – 70 ~ 80 mV, step up from + 10 mV to + 100 mV.

7. Cell Death Assay

SYTODX-Green cell death assay (Thermo Fisher Scientific) was conducted at postinduction days (PIDs) 0, 20, 25 and 30. SYTOX-Green was added to the cell medium at a concentration of 0.5 μM (1:10,000) with Hoechst (1:5000). Cells with Sytox and Hoechst were incubated in a 37 ℃ incubator for 20 minutes. Four fluorescence images were taken at 10x per well of a 96-well plate using a GE InCell Analyzer 1000. Sytox- and Hoechst-positive cells were counted using InCell Analyzer software, and percentages of cell death were calculated using the number of SYTOX-Green/Hoescht cells.

8. RNA extraction and qPCR

Total RNA for qPCR was isolated using TRIzol (Invitrogen). Briefly, the cells were lysed using TRIzol at room temperature for 5 minutes. Chloroform (0.2 ml) was added per 1 ml TRIzol and shaken vigorously. The solution was incubated at RT for 2–3 minutes followed by 15 min of centrifugation for 5 minutes at 12,000 g at 4°C. The aqueous phase was collected and transferred to a fresh microcentrifuge tube and mixed with 0.5 ml isopropanol per 1 ml TRIzol. Then, 0.5 μl of glycogen was added to the solution, followed by inversion of the tubes and incubation at -20°C for 24 hours. It was spun down for 10 minutes at 13,000 g at 4°C. The pellet was carefully washed with 1 ml 75% ethanol per 1 ml TRIzol. It was centrifuged for 5 minutes at 4°C at 13,000 g. The supernatant was carefully removed, and the pellet was resuspended in molecular grade water. The concentration was measured using a Qubit RNA quantification kit.

Equal amounts of RNA were used for cDNA. Reverse transcription was performed using SuperScript IV First Strand Synthesis SuperMix (Invitrogen, cat. # 18090050) according to the manufacturer’s protocol. qPCR was performed with fast SYBR Green PCR Master Mix (Applied Biosystems, cat. # 4385612) using the StepOne Plus Real-Time PCR System (AB Applied Biosystems, Germany). All samples were run with three technical replicates. All ct values were first normalized to the housekeeping gene HPRT1. The fold change was calculated based on the 2-ΔΔCT method.

RNA extraction for RNA sequencing was performed using the RNeasy Micro Kit (Qiagen, cat. # 74004) according to the manufacturer’s protocol. Collected RNA samples were submitted to the Genome Access Technology Center at Washington University for library preparation and sequencing.

9. RNA sequencing and analysis

Samples were prepared according to library kit manufacturer’s protocol, indexed, pooled, and sequenced on an Illumina NovaSeq 6000. Basecalls and demultiplexing were performed with Illumina’s bcl2fastq software and a custom python demultiplexing program with a maximum of one mismatch in the indexing read. RNA-seq reads were then aligned to the Ensembl release 76 primary assembly with STAR version 2.5.1a (35). Gene counts were derived from the number of uniquely aligned unambiguous reads by

Subread:featureCount version 1.4.6-p5 (36). Isoform expression of known Ensembl transcripts were estimated with Salmon version 0.8.2 (37). Sequencing performance was assessed for the total number of aligned reads, total number of uniquely aligned reads, and features detected. The ribosomal fraction, known junction saturation, and read distribution over known gene models were quantified with RSeQC version 2.6.2 (38).
All gene counts were then imported into the R/Bioconductor package EdgeR (39) and TMM normalization size factors were calculated to adjust for samples for differences in library size. Ribosomal genes and genes not expressed in the smallest group size minus samples greater than one count-per-million were excluded from further analysis. The TMM size factors and the matrix of counts were then imported into the R/Bioconductor package Limma (40). Weighted likelihoods based on the observed mean-variance relationship of every gene and sample were then calculated for all samples and the count matrix was transformed to moderated log 2 counts-per-million with Limma's voomWithQualityWeights (41). The performance of all genes was assessed with plots of the residual standard deviation of every gene to their average log-count with a robustly fitted trend line of the residuals. Differential expression analysis was then performed to analyze for differences between conditions and the results were filtered for only those genes with Benjamini-Hochberg false-discovery rate adjusted p-values less than or equal to 0.05.

For each contrast extracted with Limma, global perturbations in known Gene Ontology (GO) terms, MSigDb, and KEGG pathways were detected using the R/Bioconductor package GAGE(42) to test for changes in expression of the reported log 2 fold-changes reported by Limma in each term versus the background log 2 fold-changes of all genes found outside the respective term. The R/Bioconductor package heatmap3(43) was used to display heatmaps across groups of samples for each GO or MSigDb term with a Benjamini-Hochberg false-discovery rate adjusted p-value less than or equal to 0.05. Perturbed KEGG pathways where the observed log 2 fold-changes of genes within the term were significantly perturbed in a single-direction versus background or in any direction compared to other genes within a given term with p-values less than or equal to 0.05 were rendered as annotated KEGG graphs with the R/Bioconductor package Pathview (44).

To identify co-regulated gene networks, the Limma voomWithQualityWeights transformed log 2 counts-per-million expression data was also analyzed by weighted gene correlation network analysis with the R/Bioconductor package WGCNA (45). Briefly, all genes were correlated across each other by Pearson correlations and clustered by expression similarity into unsigned modules using a power threshold empirically determined from the data. An eigengene was then created for each de novo cluster and its expression profile was then correlated across all coefficients of the model matrix. Because these clusters of genes were created by expression profile rather than known functional similarity, the clustered gene modules were given the names of random colors where grey is the only module that has any pre-existing definition of containing genes that do not cluster well with others. These de-novo clustered genes were then tested for functional enrichment of known GO terms with hypergeometric tests available in the R/Bioconductor package clusterProfiler. Significant terms with Benjamini-Hochberg adjusted p-values less than 0.05 were then collapsed by similarity into clusterProfiler category network plots to display the most significant terms for each module of hub genes in order to interpolate the function of each significant module. The information for all clustered genes for each module were then combined with their respective statistical significance results from Limma to determine whether those features were also found to be significantly differentially expressed.

RNA sequencing analysis on SAK3-treated cells was performed using the same pipeline but with an updated version of the above programs. Total RNA integrity was determined using an Agilent Bioanalyzer or 4200 TapeStation. Library preparation was performed with 10 ng of total RNA with a Bioanalyzer RIN score greater than 8.0. ds-cDNA was prepared using the SMARTer Ultra Low RNA kit for Illumina Sequencing (Takara-Clontech) per the manufacturer's protocol. cDNA was fragmented using a Covaris E220 sonicator using peak incident power 18, duty factor 20%, and cycles per burst 50 for 120 seconds. cDNA was blunt ended, an A base was added to the 3' ends, and then Illumina sequencing adapters were ligated to the ends. Ligated fragments were then amplified for 12–15 cycles using primers incorporating unique dual index tags. Fragments were sequenced on an Illumina NovaSeq-6000 using paired end reads extending 150 bases. Base calling and demultiplexing were performed with Illumina's bcl2fastq software with a maximum of one mismatch in the indexing read. RNA-seq reads were then aligned to the Ensembl release 101 primary assembly with STAR version 2.7.9a1. Gene counts were derived from the number of uniquely aligned unambiguous reads by Subread:featureCount version 2.0.32. Isoform expression of known Ensembl transcripts was quantified with Salmon version 1.5.23. Sequencing performance was assessed for the total number of aligned reads, total number of uniquely aligned reads, and features detected. The ribosomal fraction, known junction saturation, and read distribution over known gene models were quantified with RSeQC version 4.04.

The gene count file was used to further analyze the differentially expressed genes using RUVseq (RUVr) normalization and DEseq2 with a cutoff of $\log FC \leq -1$ and $\geq 1$ and adjusted $P \leq 0.05$. We compared the upregulated genes and downregulated genes with DARs of ATAC-seq data (adjusted $P \leq 0.05$, $\log FC \leq -1$ and $\geq 1$). The DEGs and DARs were used to further analyze gene ontology terms using the IPA program.

10. Leafcutter data processing and analysis

BAM files from the RNA sequencing data were generated by STAR, using –outSAMstrandField intronMotif option (46). Samples were grouped by disease conditions and comparisons were done pairwise, as required for Leafcutter analysis. Differentially spliced clusters and introns were defined by $FDR < 0.05$, and were exported using LeafViz (47).

11. ATAC sequencing and analysis

Omni-ATAC was performed as outlined in Corces et al. (48). In brief, XDP and Ctrl fibroblasts were reprogrammed as previously described until PID 30. On PID 30, MSNs were harvested using 0.25% trypsin and a cell scraper to collect the remaining cells. Each sample was treated with DNase for 30 minutes before collection. Approximately 50,000 cells were collected for library preparation. The transposition reaction was completed with Nextera Tn5 Transposase (Illumina Tagment DNA Enzyme and Buffer Kit, Illumina) for 30 minutes at 37 °C, and library fragments were amplified under optimal amplification conditions. Final libraries were purified by the DNA Clean & Concentrator 5 Kit (Zymo). Libraries were sequenced on an Illumina NovaSeq S4 XP (Genome Technology Access Center at Washington University).

ATAC-seq analysis in directly reprogrammed neurons was performed as previously described (49) by using the ATAC-seq Integrative Analysis Package (AIAP) pipeline (50). The ATAC-seq FASTQ files were trimmed by Cutadapt and aligned to the reference genome by BWA. ATAC peaks were called by MACS and used to obtain read counts. Differential peaks were identified using RUVseq (RUVr) normalization and then DEseq2 with a cutoff of logFC ≤-1 and ≥ 1 and adjusted P ≤ 0.05. Gained peaks were defined as open (more accessible) chromatin regions. Conversely, all reduced peaks were defined as closed chromatin regions. We annotated genes nearest to open or closed regions using chipseeker and compared them with DEGs of RNA-seq data (adjusted P ≤ 0.05, logFC ≤ -1 and ≥ 1).

### Results

## Medium spiny neurons directly reprogrammed from XDP patient fibroblasts show mature neuronal and MSN markers

To assess the reprogramming efficiency of fibroblasts from affected XDP patients, we carried out the direct conversion of fibroblast samples from three independent XDP patients and age- and sex-matched control fibroblasts from three healthy individuals (30–55 years old) (Supp Table 1). We used miR-9/9*-124 and *CTIP2*, *DLX1, DLX2* (as *DLX1*-P2A-*DLX2* construct), and *MYT1L* (CDM) transcription factors that guide neuronal conversion to medium spiny neurons (MSNs) (Fig. 1A). The reprogramming of XDP fibroblasts (XDP-FBs) to MSNs (XDP-MSNs) was confirmed using positive immunostaining for neuronal markers MAPT and TUBB3 (Fig. 1B, C, Supp Fig. 1A, B) and MSN marker, DARPP-32 (Fig. 1C, Supp Fig. 1A). Quantification of TUBB3 and DARPP32 staining in 3 independent lines of Ctrl-MSNs and 3 independent patient lines of XDP-MSNs showed more than 90% positive cells over the total nucleus counted (DAPI) (Fig. 1D). There is no statistically significant difference in expression of TUBB3 or DARPP32 between Ctrl- and XDP-MSNs.

The reprogramming of MSNs was confirmed by RNA sequencing of starting fibroblasts and MSNs. RNA sequencing showed a battery of neuronal and MSN marker genes selectively enriched in reprogrammed MSNs (Fig. 1E). Fibroblast genes such as *ITGB1* were successfully erased in reprogrammed MSNs, consistent with previous reports, while neuronal genes such as *SNAP25* and *PPP1R1B*, an MSN marker, were enriched only in reprogrammed MSNs (Supp Fig. 1C). The expression of long genes defined by genes more than 150 kb in length (from genomic START to STOP codons), a transcriptomic feature unique to neurons(51–53), was found to be significantly increased compared to fibroblasts, demonstrating the neuronal identity acquired in reprogrammed cells (Fig. 1F).

RNA sequencing validation was done by qPCR for neuronal and MSN marker gene expression. *MAP2* and *NEUN* are pan-neuronal markers. *nTAF1* is an isoform of *TAF1* that is specific to neurons (2, 54). *SRRM4* is a neuron-specific splicing factor (55). *PPP1R1B* is a marker specific to MSNs. These markers were significantly enriched similarly for both XDP and Ctrl reprogrammed MSNs compared to starting fibroblasts (Fig. 1G). As a measurement of neuronal identity, we assessed the membrane excitability of reprogrammed XDP- and Ctrl-MSNs by whole-cell recording. The reprogrammed MSNs showed voltage-dependent inward currents as well as action potentials (APs) upon current injections. All cells patched fired action potential (AP) with around 71% of cells firing multiple APs in Ctrl-MSNs (total # cells patched = 52) and around 76% cells firing multiple APs in XDP-MSNs (total # cells patched = 68) (Fig. 1H; right panel = representative APs and inward currents). Taken together, these results show that direct neuronal reprogramming by miR-9/9*-124 and CDM factors successfully generate MSNs from fibroblasts of XDP patients.

## Patient-derived XDP-MSNs show spontaneous neurodegeneration

XDP is characterized by degeneration of MSNs in the striatum, therefore we assessed whether reprogrammed XDP-MSNs would exhibit similar neurodegenerative phenotypes as seen in patient brains. The SYTOX-Green assay, which marks cells undergoing cell death, indicated no significant difference between XDP and Ctrl fibroblasts postinduction day (PID) 0. However, as reprogramming cells acquire the identity of MSNs at PID 30 and beyond, XDP-MSNs from three independent XDP patients showed a significantly increased level of cell death compared to Ctrl-MSNs from three age-matched healthy individuals, as indicated by the number of SYTOX-Green signals over Hoescht signals (Fig. 2A). It is worth noting that XDP-MSNs underwent spontaneous cell death without adding extrinsic insults, suggesting that cell-intrinsic properties underlie the increased vulnerability to neurodegeneration in XDP-MSNs. A phenotype that accompanies neurodegeneration is DNA damage. As shown in Fig. 2B, we found that XDP-MSNs exhibited significantly increased levels of DNA damage, as marked by the positive staining of the DNA double-stranded break marker 53BP1. By quantifying the number of DNA breaks per cell, we found that XDP-MSNs contained a significantly higher number of DNA double-stranded breaks. 8OHdG, a marker of oxidative damage, was also significantly increased in XDP-MSNs (Fig. 2C). To substantiate the relationship between cell death and DNA damage observed in XDP-MSNs, we tested whether blocking the cell death pathway induced by DNA damage would alter the cell death level in XDP-MSNs. Treating XDP-MSNs with KU60019, an inhibitor of ATM kinase that promotes cell death in response to DNA damage (18), rescued XDP-MSNs from cell death close to the control level (Fig. 2D), suggesting the interplay between DNA damage seen in XDP-MSNs and neuronal death. Altogether, we found that XDP-MSNs can capture cell-intrinsic properties underlying XDP-associated neurodegeneration.

## Patient-derived XDP-MSNs show increased vulnerability to TNFα

We also tested whether XDP-MSNs would be hypersensitive to additional cellular insults. One such insult is TNFα, which is a pro-inflammatory molecule and can lead to cellular apoptosis (56). In XDP fibroblasts as well as XDP neuronal stem cells (NSCs), the NFκB pathway which is activated by TNFα has been shown to be mis regulated (57). We treated XDP-MSNs with TNFα to test whether TNFα would exacerbate neurodegeneration. We observed that when the cells were treated with increasing doses of TNFα, XDP-MSNs showed significantly increased cell death levels, while the same concentration of TNFα did not induce neuronal death in Ctrl-MSNs compared to the PBS control (Fig. 2E). These results suggest that XDP-MSNs showed selective sensitivity to TNFα compared to Ctrl-MSNs. We also assessed mitochondrial dysfunction as a degeneration-associated phenotype. Cytochrome c is a mitochondrial protein that translocates to the nucleus when cells have dysfunctional mitochondria and/or are undergoing apoptosis (58, 59). Quantification of the percentage of cytochrome c-positive nuclei in Ctrl vs XDP-MSNs showed that XDP-MSNs treated with TNFα showed a significantly increased cytochrome c signal in the nucleus, whereas Ctrl-MSNs remained unaffected with or without TNFα treatment (Fig. 2F). Therefore, our results indicate that XDP-MSNs from multiple independent patients can reliably recapitulate increased susceptibility to cellular stress at doses that do not affect control cells.

## Transcriptomic and chromatin landscape analysis of XDP-MSNs reveals dysregulation in pathways involved in neurodegenerative diseases

To gain further insights into the differential cellular state between XDP-MSNs and Ctrl-MSNs, we carried out comparative RNA-seq and ATAC-seq analyses to determine whether any potential differences in transcriptome and chromatin accessibilities would exist between XDP and Ctrl-MSNs (Fig. 3A, 4A). Both RNA and ATAC-seq were performed on PID 28. For RNA-seq analysis, differentially expressed genes (DEGs) that met the cutoff of logFC ± 1 with adj P-Val ≤ 0.05 were considered significant (Supp Table 3). The comparison between XDP- and Ctrl-MSNs resulted in 239 significantly upregulated and 463 significantly downregulated genes in XDP-MSNs (Fig. 3B). GO analysis was performed for the down or up regulated genes in XDP and that resulted in several terms associated with neuronal health such as “Regulation of response to stress”, “Synaptic transmission, GABAergic” (Fig. 3C). Figure 3D shows representative IGV tracks of genes that are significantly downregulated (*TDO2*) or significantly upregulated (*PAX3*) in XDP-MSNs.

Further, weighted gene co-expression network analysis (WGCNA) was performed with the RNA-seq datasets to identify correlation between gene expression in XDP-MSNs with gene networks that cluster with those genes. WGCNA resulted in 5 modules that were significantly enriched in XDP-MSNs (p-value cutoff ≤ 0.05) compared to Ctrl-MSNs (Fig. 3E, Supp Table 4, 5). One of the significant modules was the “salmon” module with a positive correlation coefficient in the XDP-MSNs. This module included 919 genes (Supp Table 5) that were implicated in pathways such as splicing, autophagy, and mitophagy (Fig. 3G). Interestingly, the genes from this module were enriched for putative binding sites for several transcription factors with TAF1 being the most significant as analyzed by Enrichr (Fig. 3F). This is an interesting finding for XDP specifically because the mutations associated with the disease are around TAF1 gene. TAF1 as the top enrichment TF is also observed in another study looking into proteomic analysis of XDP neuronal stem cells, or MSNs derived from induced pluripotent stem cells (60).

In addition, regulation of alternative splicing of genes have been implicated in neurodegenerative diseases as well as repeat expansion diseases (61). Previous studies have also shown that in XDP, the SVA insertion in intron 32 of the *TAF1* gene leads to intron retention, suggesting that splicing alteration may occur in XDP (4, 62). To explore the alternative splicing differences in XDP-MSNs compared to Ctrl-MSNs, leafcutter analysis was performed which resulted in 30 genes that are significantly differentially splicing in XDP-MSNs (adjusted P ≤ 0.05) (Supp Table 6). Supp Fig. 2A shows representative splicing tracks in XDP-MSNs compared to Ctrl-MSNs. Among these genes, *GTF2H2, GAS5, ARF4, RNF212* were detected top significant genes that showed alternative splicing differences in XDP compared to Ctrl-MSNs. Interestingly, KEGG pathway analysis shows that the genes that show alternative splicing in XDP-MSNs were implicated in other neurodegenerative diseases such as ALS, PD, HD and AD (Supp Fig. 2B). Notably, similar to the WGCNA results of the salmon module (Fig. 3), the differentially alternatively spliced genes were also enriched for putative TAF1 binding sites (Supp Fig. 2C). GO analysis reveals mRNA splicing and RNA binding as some of the most significant terms (Supp Fig. 2D-F).

In addition to transcriptome analyses, chromatin accessibility was measured by ATAC-sequencing (48) (Fig. 4A). Differentially accessible regions (DARs) that met the cutoff of logFC ± 1 with adj P-Val ≤ 0.05 were considered significant (Supp Table 7). XDP-MSNs had 1632 regions that were significantly open and 8040 regions that were significantly closed compared to Ctrl-MSNs. The heatmap in Fig. 4B shows open and closed regions that are within ± 2 kb of a gene transcription start site (TSS). Next, the significant regions/DARs (1632 open and 8040 closed) were annotated for genes within ± 2 kb of TSS that corresponded to that region using chipseeker. The results were filtered for duplicated genes (genes with multiple DARs) as well as the distal intergenic regions. This resulted in 4045 genes that had significant open or closed DARs in XDP-MSNs. The 4045 genes corresponding to the significant DARs were implicated in axonal guidance signaling and synaptogenesis among the pathways identified (Fig. 4C). When we integrated the significant gene lists from RNA-seq and ATAC-seq datasets, 200 genes were upregulated or downregulated DEGs, containing opened or closed DARs (Fig. 4D). Examples of genes that are downregulated and closed (*RNF212*) or upregulated and open (*DDX43*) in XDP-MSNs are shown in Fig. 4E. Pathway analyses for these 200 genes were significantly enriched with functional pathways such as axonal guidance signaling and, more interestingly, calcium signaling (Fig. 4F).

## SAK3, a T-type calcium channel enhancer, rescues XDP-MSN cell death

As shown in Fig. 4, molecular function analysis of DEGs and DARs in XDP-MSNs suggested dysfunction in neurodegeneration as well as calcium signaling pathways. We then further investigated the possibility of therapeutic compounds that have been evaluated in other neurodegenerative diseases to validate our transcriptomic findings and to establish this model as a way to understand XDP disease. SAK3 is an activator of the T-type calcium channel (30). SAK3 has been shown to be beneficial in other neurodegenerative diseases, as well as in a TAF1-deficient animal model (32–34). Importantly, when XDP and Ctrl-MSNs were treated with SAK3, we observed that XDP-MSNs were significantly rescued from cell death in a dose-dependent manner compared to DMSO (Fig. 5A). This protective effect of SAK3 was detected in XDP-MSN lines from three independent XDP patients where the cell death of XDP-MSNs, initially approximately 40%, was reduced to a level similar to that of Ctrl-MSNs. Furthering this finding, we also measured the effect of SAK3 on DNA damage and mitochondrial dysfunction by quantifying DNA double stranded break with 53BP1 and cytochrome c in the nucleus, respectively. SAK3 led to reduction in the DNA damage level in XDP-MSNs (Fig. 5B). Also, SAK3 lowered cytochrome c translocation to the nucleus in XDP-MSNs suggesting improvement in mitochondrial integrity (Fig. 5C). Interestingly, the exacerbation of neurodegenerative phenotypes by TNFα as a cellular insult can be also resolved with the use of SAK3 in XDP-MSNs including the rescue of exacerbated cell death with SAK3 (Fig. 5D), and lowering of the cytochrome c translocation in the nucleus (Fig. 5E). Therefore, our results demonstrate the neuroprotective effect of SAK3 in patient-derived XDP-MSNs.

To further understand the mechanism of how SAK3 plays a role in rescuing cell death of XDP-MSNs, we performed transcriptomic analysis of XDP and Ctrl-MSNs treated with SAK3 or DMSO as a negative control. Interestingly, we found that when Ctrl-MSNs treated with SAK3 vs DMSO were compared, there were 3 significant DEGs, whereas XDP-MSNs treated with SAK3 vs DMSO resulted in 450 significant DEGs (Fig. 6A, Supp Table 8). Figure 6B shows representative IGV tracks of *SNAP25*, a neuronal gene that is not altered in XDP or with SAK3 treatment indicating the neuronal identity persists with SAK3 treatments, as well as an example of a gene *PTAFR*, a neurodegeneration and inflammation associated gene (63, 64) whose expression is upregulated in XDP but reduced by SAK3 treatment. *EDN2*, another gene that has been studied in the context of retinal degeneration (65), is downregulated in XDP-MSNs whereas SAK3 treatment increases its expression. Gene enrichment analysis of those 450 DEGs using Ingenuity Pathway Analysis revealed several pathways that were common with DEGs of SAK3 compared to DMSO. Synthesis of reactive oxygen synthesis was predicted to be downregulated with SAK3 (Fig. 6C) and the genes were also enriched for Ion homeostasis of cells (Fig. 6C). Furthermore, 55 genes that were differentially expressed in XDP-MSNs changed their expression directionality with SAK3 treatment toward the expression in Ctrl-MSNs (Fig. 6D). Gene enrichment analysis for these genes using KEGG and Gene Ontology analysis revealed pathways such as protein digestion and absorption, and cAMP signaling pathway (Fig. 6E). Taken together, these results indicate that the neuroprotective effect of SAK3 is characterized by gene expression changes including those DEGs in XDP-MSNs reversing towards healthy control MSNs.

## Discussion

Over the past four decades, significant strides have been made in the field of XDP research, shedding light on various aspects of the disorder, such as its pathogenesis, the role of *TAF1* mutations, and transcriptomic differences, among others (66). However, despite these advancements, there has been no model system that accurately captures the haplotype, age of onset, and neurodegenerative changes observed in the brains of XDP patients. The present study represents a critical demonstration of modeling neurodegeneration of XDP using patient-derived neurons that enable investigations of the adult-onset cellular phenotypes of XDP in MSNs. To achieve this, a direct reprogramming method was utilized to convert XDP patient fibroblasts into MSNs, effectively recapitulating the genetic and chromatin dysregulation of XDP. In addition to the examination of XDP phenotypes, these reprogrammed XDP-MSNs can be harnessed to test therapeutic candidates that alleviate the neurodegeneration phenotypes.

We demonstrate that fibroblast samples are amenable to direct neuronal reprogramming approach results in the successful conversion of adult patient fibroblasts into mature MSNs, providing a robust model for studying XDP. XDP-MSNs exhibited significantly higher rates of cell death and phenotypes associated with neurodegeneration, including DNA damage and mitochondrial dysfunction. Previous research has indicated dysregulation in the DNA mismatch pathway genes in XDP (67), but direct evidence was shown in our study, as the increase in cell death observed in XDP-MSNs was found to be reversible through the blockade of the ATM/ATR kinase pathway, which plays a crucial role in DNA damage-induced cell death. Notably, our observations indicate a pronounced vulnerability of MSNs in XDP to cellular stress, particularly evidenced by a substantial increase in cell death when exposed to TNFα, an inflammatory cytokine known to activate the NFκB pathway. This finding is particularly significant, as the downregulation of the NFκB pathway and an increase in several proinflammatory markers have been identified as contributing factors in XDP pathogenesis (57, 68). The dysregulation of NFκB as well as the involvement of TNFα has been studied in the context of other neurodegenerative diseases such as Parkinson's Disease (PD), where there is an enhancement in the levels of TNFα in the striatum (69).

Our results indicate significant transcriptomic and chromatin differences between XDP-MSNs and Ctrl-MSNs, with these differences arising as a consequence of the MSN cell fate. These chromatin and transcriptomic variations observed in XDP-MSNs are associated with several pathways that have connections to other neurodegenerative diseases, including the calcium signaling pathway (70). Moreover, a recent study looking at aging in directly reprogrammed medium spiny neurons from longitudinally collected fibroblasts from healthy individuals identified dysregulation of the calcium signaling pathway (26). Therefore, restoring dysregulation of calcium signaling could be a concept that could be applied to age-associated onset of neurodegeneration. As such, the results of our study offer evidence that cellular pathways altered in XDP-MSNs can be restored by SAK3 to alleviate the neurodegeneration phenotype in XDP-MSNs. SAK3, a T-type calcium channel activator has previously been studied in the context of other neurodegenerative disorders and in a mouse model of *Taf1* knockdown (31, 33, 34, 71–73). SAK3 has also been shown to rescue behavioral phenotypes in a rat model of *Taf1* intellectual disability syndrome (33). Another study has indicated that the rescue of phenotype by SAK3 is due to improved proteosome activity that is reduced in AD (73). Encouragingly, treatment with SAK3 successfully ameliorated cell death and mitochondrial dysfunction in XDP-MSNs. Furthermore, we observed a distinct set of genes whose directionality changed in XDP-MSNs following SAK3 treatment, where the genes that respond to the SAK3 treatment have implications in neurodegeneration as implicated previous studies. For example, *PTAFR* is a proinflammatory gene involved in Alzheimer's disease (63) and is upregulated in XDP, but the expression level is reduced by SAK3 treatment. *EDN2*, a receptor that has been shown to be neuroprotective in retinal neurodegeneration (65) is downregulated in XDP-MSNs, while SAK3 treatment rescued the expression of *EDN2*. In addition, transcriptomic analysis with SAK3 treatment shows reduction of oxidative stress which is a validation of the phenotype rescue that was seen in SAK3 treated MSNs. These findings reflect the protective effect of SAK3 in XDP-MSNs from phenotype to transcriptomic changes.

### Conclusions

Our understanding of XDP's causal factors had been limited to prior discoveries highlighting variable expression of TAF1 isoforms (2, 3, 54, 74), the pathogenic effect of SVA insertion (5, 75), or DNA repair genes (67). However, our study has now validated the presence of additional genes that differ in XDP-MSNs and can be modulated by SAK3 that protects XDP-MSNs from neurodegeneration. Further investigations are warranted to elucidate the roles of genes altered in XDP-MSNs and how they may be targeted as potential therapeutic strategies for individuals affected by XDP.

### Declarations

#### Ethics approval and consent to participate:

Not applicable

#### Consent for publication:

Not applicable

## Competing interests:

The authors declare that they have no competing interests.

## Funding:

The funding for this study was provided by the grant and awards to ASY: Collaborative Center for XDP (Agreement Reference #: 239295), RF1AG056296 (NIH/NIA), R01NS107488 (NIH/NINDS).

## Authors' contributions:

SA and ASY designed the study. SA and SC conducted the experiments. YY performed the electrophysiology experiments. WKK provided resources for sequencing data analysis. SA performed data analysis. KFB performed WGCNA analysis. KFB and LSC performed the leafcutter analysis. SA and ASY wrote the manuscript. DCB provided scientific insights throughout the project and edited the manuscript. ASY supervised the overall project.

## Acknowledgements:

We thank the Genome Technology Access Center at the McDonnell Genome Institute at Washington University School of Medicine for help with genomic analysis. The Center is partially supported by NCI Cancer Center Support Grant #P30 CA91842 to the Siteman Cancer Center. This publication is solely the responsibility of the authors and does not necessarily represent the official view of NCRR or NIH.

## Availability of data and materials:

The datasets generated and/or analyzed during the current study will be available in the GEO repository.

## Figures

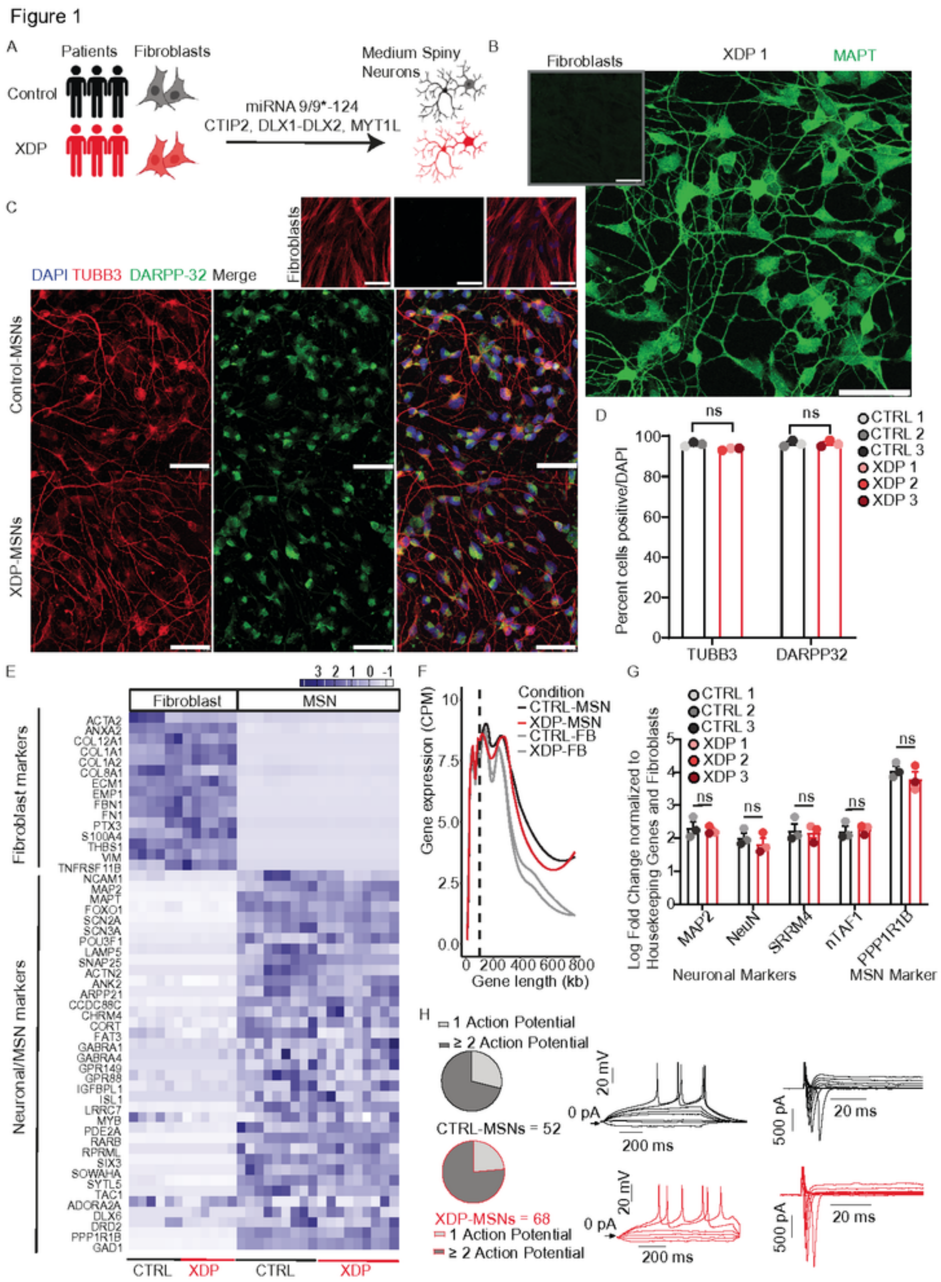

### Figure 1

**Medium spiny neuron conversion from XDP human dermal fibroblasts using microRNAs and striatal factors.**

A). Graphical abstract showing the direct reprogramming method using miR9/9*-124 and striatal factors *CTIP2*, *DLX1-P2A-DLX2*, and *MYT1L*. Three XDP patient cell lines are represented in red, and 3 age-and sex-matched control cell lines are represented in black.

B). Representative images showing starting fibroblasts and directly reprogrammed XDP-MSN patient-1 (XDP-1) immunostained for MAPT. The scale bar represents50 µm.

C). Representative images showing starting XDP fibroblasts and directly reprogrammed Ctrl and XDP-MSNs immunostained for DAPI (blue), TUBB3 (red), DARPP-32 (green), and all channels merged. The scale bar represents50 µm.

D). Quantification of TUBB3 and DARPP-32 immunostaining in 3 control and 3 XDP patient cell lines. Number of cells counted per cell line: Ctrl-1= 152, Ctrl-2= 170, Ctrl-3= 127, XDP-1= 142, XDP-2= 146, XDP-3= 170.

E). Heatmap of fibroblast and neuronal gene expression in starting fibroblasts compared to the directly reprogrammed Ctrl and XDP-MSNs. Fibroblasts: N = 3-XDP and 3-Ctrl lines with 2 technical replicates each. MSNs: N = 3-XDP and 3-Ctrl lines with 3 technical replicates each.

F). Long gene expression is increased in Ctrl-MSNs (black) and XDP-MSNs (red) compared to starting fibroblasts (gray).

G). Expression of neuronal and MSN-specific genes in both Ctrl and XDP-MSNs normalized to starting fibroblasts and housekeeping gene *HPRT1*. Paired t-test, ns = nonsignificant.

H). Graphs showing the percent action potential in Ctrl-MSNs and XDP-MSNs. Representative traces of action potentials, Inward INa, and outward IK currents from Ctrl-MSNs and XDP-MSNs. Current-clamp recordings of evoked action potentials (APs) with progressive current-injection steps.

Figure 2

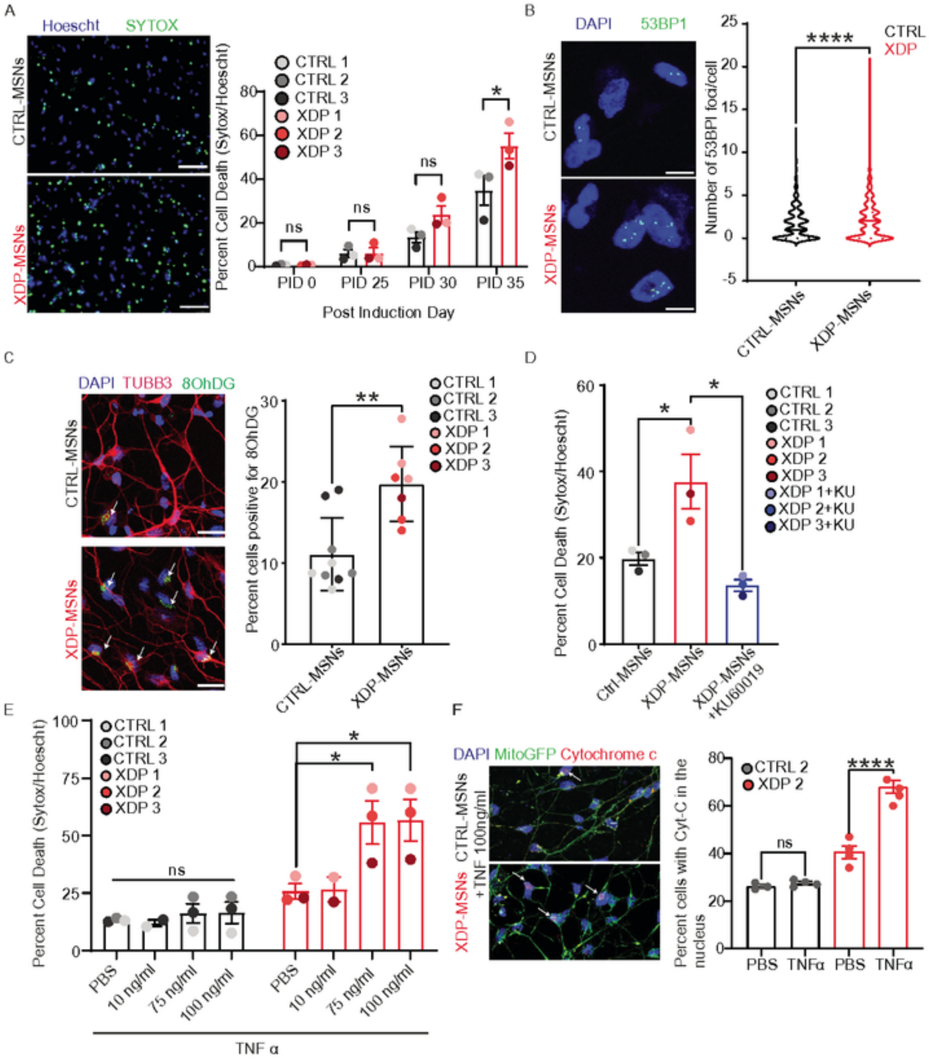

### Figure 2

**XDP patient-derived cells show a spontaneous neurodegenerative phenotype and increased vulnerability to cellular insults.**

A). SYTOX assay as a cell death marker in XDP-MSNs compared to Ctrl-MSNs. The left panel shows representative images of SYTOX staining for Ctrl- and XDP-MSNs. The scale bar represents 50 μm. N = 3

patient cell lines per condition (3-XDP, 3-Ctrl) with 8-10 replicates each. Paired t-test, *$p<0.05$, ns = nonsignificant.

B). Representative images of 53BP1 immunostaining, marking DNA double-stranded breaks. The scale bar represents 10 μm.The graph shows the number of 53BP1 foci/cell for a total of 1560 cells counted/condition from 3 individual XDP patient cell lines and 3 Ctrl cell lines. Unpaired t-test, ****$p<0.0001$.

C). Representative images of 8OHdG, a marker of oxidative DNA damage. The scale bar represents 25 μm. The graph shows the percentage of cells that are positive for 8OHdG in Ctrl and XDP-MSNs. N = 3 patient cell lines per condition (3 XDP, 3 Ctrl) with 4-12 replicates each. Unpaired t-test, **$p<0.01$.

D). SYTOX analysis showing cell death in XDP-MSNs with an inhibitor of ATM kinase (KU-60019). N = 3 patient cell lines per condition (3 XDP, 3 Ctrl). Welch’s t test, ****$p<0.0001$

E). Dose-response SYTOX assay to compare XDP-MSNs to Ctrl-MSNs with and without the addition of TNFα for 36 hours at PID 25. One-way ordinary ANOVA with multiple comparisons. *p-adj < 0.05.

F). Representative image shows immunofluorescence staining for DAPI (nucleus), MitoGFP (mitochondria), and Cytochrome c staining in TNFα (100 ng/ml) treated MSNs. The scale bar represents 25 μm. The graph shows the percentage of cells with cytochrome c in the nucleus in Ctrl and XDP-MSNs treated with PBS or TNFα (100 ng/ml). N = 1 Ctrl and 1 XDP patient cell lines. One-way ANOVA with multiple comparisons. ****p-adj<0.0001.

Figure 3

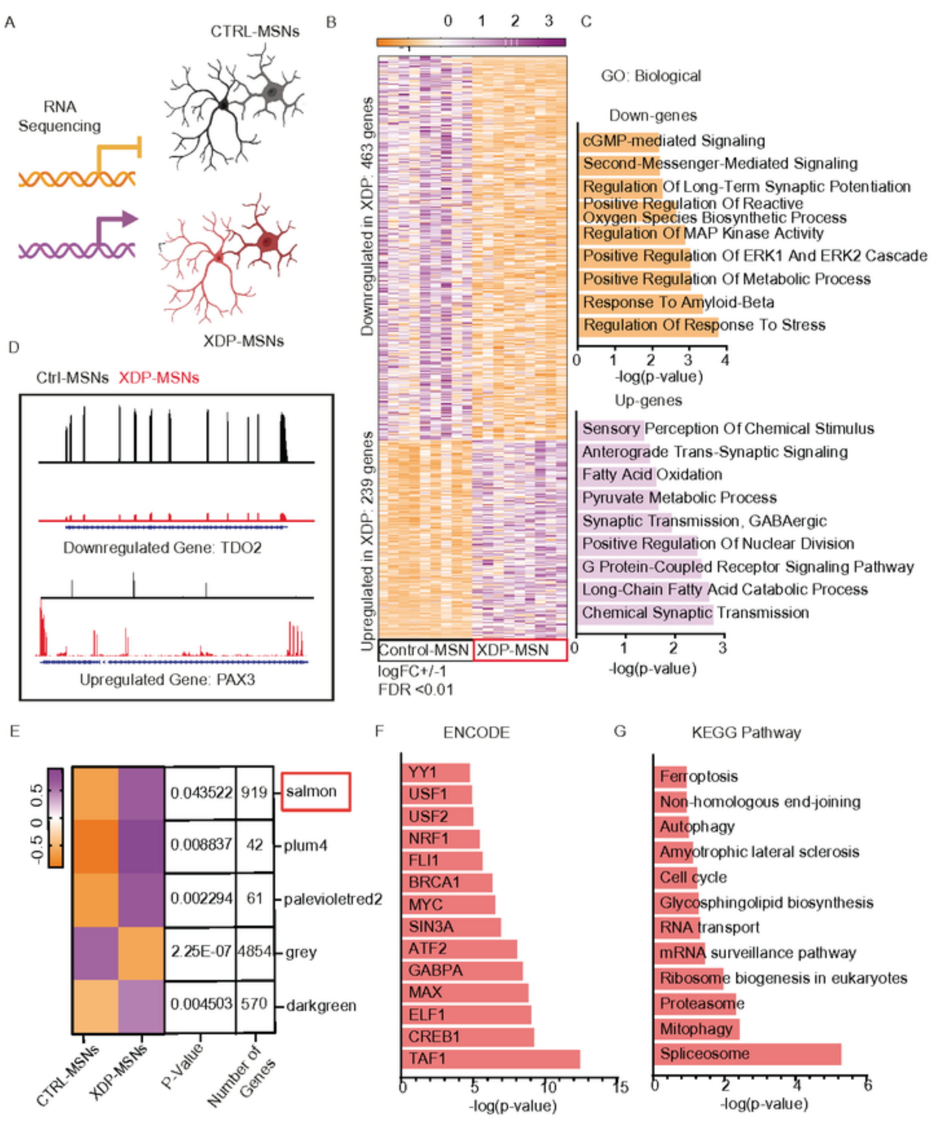

### Figure 3

**Differentially expressed genes between Ctrl and XDP-MSNs show neurodegeneration-associated terms.**

A). Graphical abstract showing the RNA sequencing method in Ctrl and XDP-MSNs.

B). Heatmap showing differentially expressed genes (DEGs) in Ctrl and XDP-MSNs using RNA sequencing (adj P-Val≤=0.05, logFC≤=1 or ≥=1).

C). GO: Biological analysis of downregulated genes in XDP-MSNs (orange). GO: Biological analysis of upregulated genes in XDP-MSNs (lavender).

D). Gene expression tracks *TDO2*, a gene that is downregulated in XDP-MSNs, and *PAX3*, a gene that is upregulated in XDP-MSNs using IGV.

E). Five significant modules from WGCNA analysis, the associated p-values, and the total number of genes included in the module.

F). ENCODE analysis using the genes from the Salmon module. *TAF1* is the top gene from the analysis.

G). KEGG pathway analysis of the Salmon module resulted in several terms that are significantly associated with the list of genes.

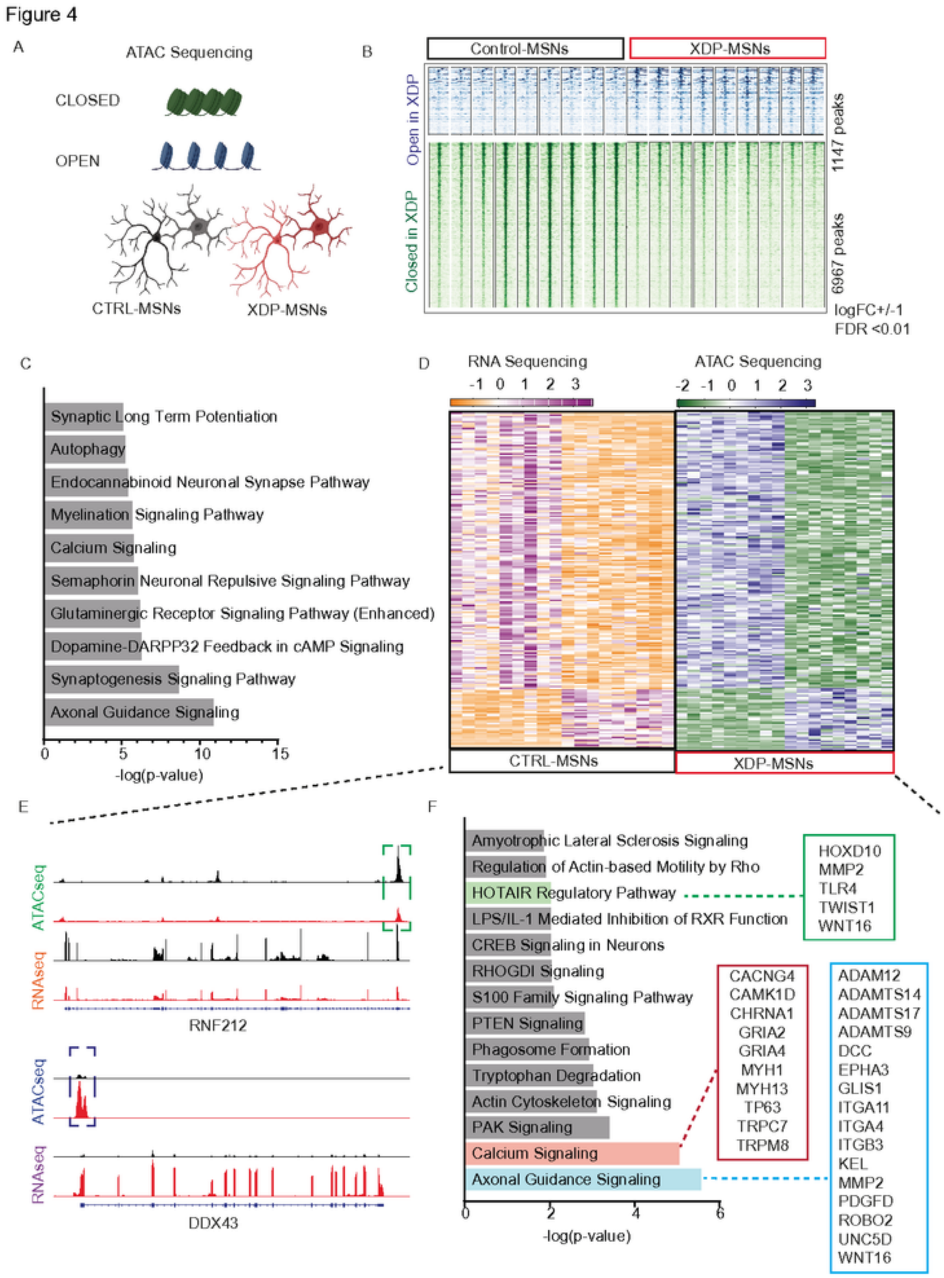

### Figure 4

**Differentially expressed genes and differentially accessible regions between Ctrl and XDP-MSNs show neurodegeneration-associated terms.**

A). Graphical abstract showing ATAC sequencing, the chromatin profiling method in Ctrl and XDP-MSNs.

B). ATAC sequencing shows more than 8000 differentially open or closed regions in XDP-MSNs compared to Ctrl-MSNs (adj P-Val≤=0.01, logFC≤=1 or ≥=1).

C). GO analysis of open or closed chromatin regions in XDP-MSNs.

D). ATAC sequencing resulted in more than 4000 genes that had one or more differentially accessible regions (DARs) in XDP-MSNs compared to Ctrl-MSNs, out of which 200 genes overlapped with differentially expressed genes from RNA sequencing. Heatmaps showing XDP and Ctrl-MSN differences in ATAC sequencing (left) and RNA sequencing (right).

E). Representative gene tracks in IGV that are both downregulated and closed in XDP-MSNs (*RNF212*) and upregulated and open in XDP-MSNs (*DDX43*).

F). IPA analysis ofthe overlapping gene dataset shows pathways that are disrupted in XDP-MSNs, including calcium signaling and axonal guidance signaling, and the genes associated with the terms.

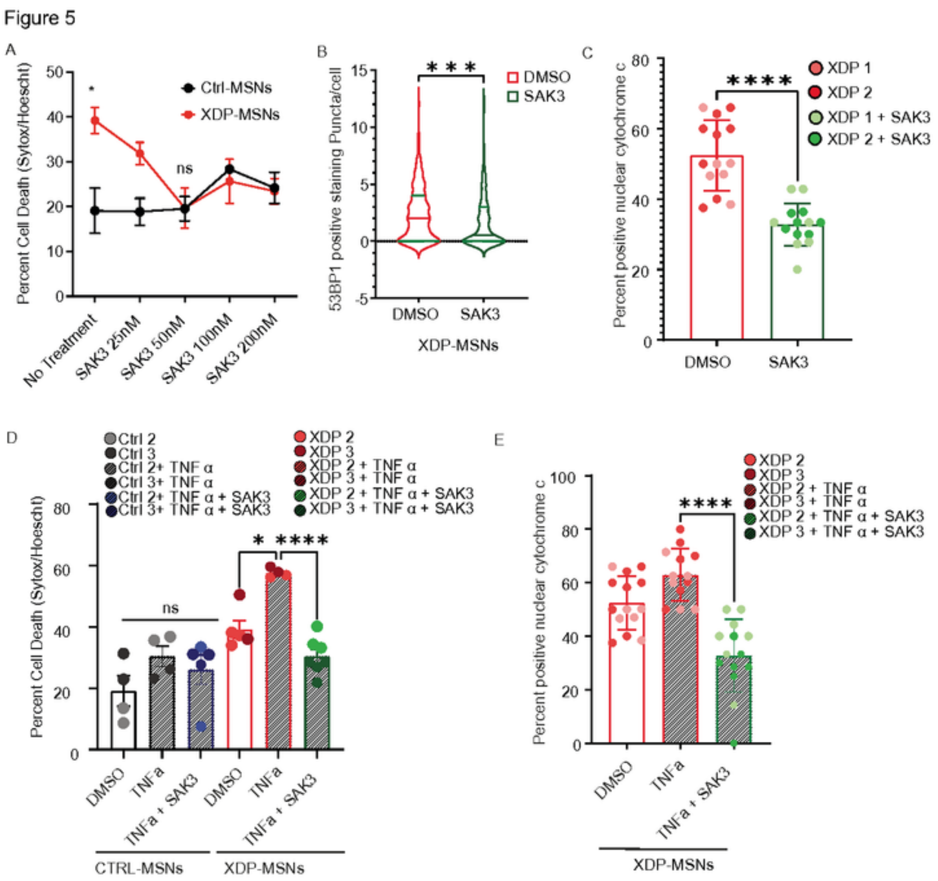

### Figure 5

#### SAK3 has a protective effect on neurodegeneration phenotypes of XDP-MSNs.

A). Dose-response curve of SAK3 at no treatment, 25 nM, 50 nM, 100 nM, and 200 nM in Ctrl and XDP-MSNs at PID 30. N = 3 patient cell lines per condition (3-XDP, 3-Ctrl) with 8-10 replicates each. Welch's t-test, ****p<0.0001, ns = nonsignificant.

B). DNA double-stranded breaks quantification in XDP-MSNs with and without SAK3 using 53BP1 immunostaining. N = 3 patient cell lines per condition (3-XDP, 3-Ctrl) with ~500 cells counted. Unpaired t-test, ***$p<0.001$.

C). Mitochondrial dysfunction as measured by cytochrome c translocation. N = 2 patient cell lines per condition (2-XDP) with 8-10 replicates each. Unpaired t-test, ****$p<0.0001$.

D). SYTOX assay showing percent cell death for Ctrl- and XDP-MSNs treated with TNFα and/or SAK3. N = 2 patient cell lines per condition (2-XDP) with 2-4 replicates each. One-way ANOVA with multiple comparisons, *$p<0.05$, ****$p<0.0001$, ns = nonsignificant.

E). Mitochondrial dysfunction as measured by cytochrome c translocation. N = 2 patient cell lines per condition (2-XDP) with 8-10 replicates each. One-way ANOVA with multiple comparisons, ****$p<0.0001$.

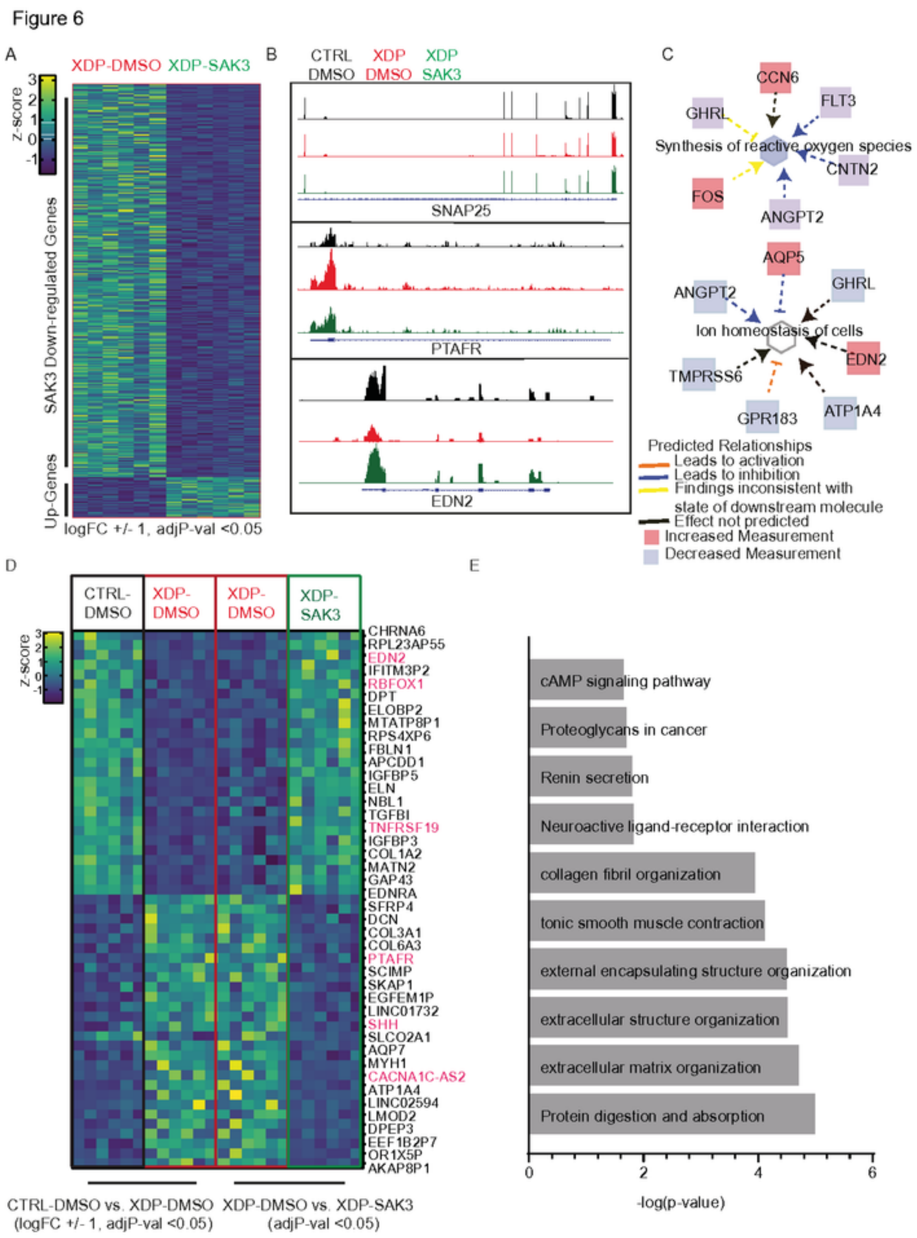

### Figure 6

**SAK3 alters the XDP-MSNs transcriptome to resemble the transcriptome of Ctrl-MSNs.**

A). Heatmap of 450 genes down or up regulated with SAK3 treatment vs. DMSO in XDP-MSNs. The genes are a result of filtering for adj P-Val≤=0.05, logFC≤=1 or ≥=1.

B). Representative gene expression tracks. *SNAP25*, a negative control gene that is expressed similarly in all three conditions. *PTAFR*, a gene that is overexpressed in XDP-MSNs, but the expression is stabilized with SAK3 treatment. *EDN2*, which is downregulated in XDP-MSNs is rescued with the use of SAK3.

C). IPA analysis ofthe 450 genes shows pathways involved with SAK3 treatment of XDP-MSNs.

D). Heatmap showing a subset of DEGs that change the direction with SAK3. The 55 genes overlap between DEGs from Ctrl-DMSO compared to XDP-DMSO, with DEGs from XDP-DMSO compared to XDP-SAK3 but follow opposite directions of expression.

E). KEGG and GO analysis of 55 genes that change direction in XDP-SAK3 towards CTRL-DMSO.

## Supplementary Files

This is a list of supplementary files associated with this preprint. Click to download.

- AryalFigures31024opt7.png
- AryalFigures31024opt8.png
- AryalSupplementalTable31024.xlsx
